# Ion migration drives self-passivation in perovskite solar cells and is enhanced by light soaking†

**DOI:** 10.1039/d1ra01166a

**Published:** 2021-03-25

**Authors:** Bart Roose

**Affiliations:** Department of Physics, Cavendish Laboratory, University of Cambridge 19 JJ Thomson Avenue Cambridge CB3 0HE UK br340@cam.ac.uk

## Abstract

Perovskite solar cells have rapidly become the most promising emerging photovoltaic technology. This is largely due to excellent self-passivating properties of the perovskite absorber material, allowing for a remarkable ease of fabrication. However, the field is plagued by poor reproducibility and conflicting results. This study finds that dynamic processes (ion migration) taking place after fabrication (without external stimuli) have a large influence on materials properties and need to be controlled to achieve reproducible results. The morphological and optoelectronic properties of triple cation perovskites with varying halide ratios are studied as they evolve over time. It is found that ion migration is essential for self-passivation, but can be impeded by low ion mobility or a low number of mobile species. Restricted ion movement can lead to crack formation in strained films, with disastrous consequences for device performance. However, a short light soaking treatment after fabrication helps to mobilize ions and achieve self-passivation regardless of composition. The community should adopt this treatment as standard practice to increase device performance and reproducibility.

## Abstract 2

Ion migration can assist self-passivation and strain relaxation in lead halide perovskite films, while restriction of ion migration can lead to crack formation. Light soaking increases ion migration, allowing self-passivation and strain relaxation.

## Graphical abstract

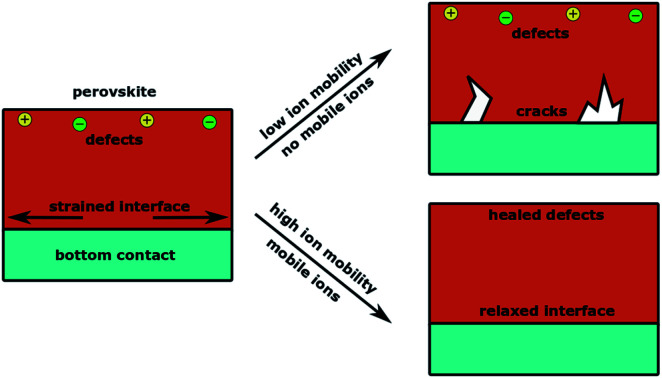

## Introduction

Perovskite solar cells (PSCs) have rapidly achieved power conversion efficiencies (PCEs) comparable to established silicon based solar cells.^1^ Lead-halide perovskites exhibit long charge diffusion lengths, high absorption coefficient, compositional tunability, ease of processing and high tolerance to defects, which makes them ideal materials for solar cells.^2^ Efforts are now being made to push PSCs towards their theoretical limits,^3^ where an important role is reserved for defect passivation. Defects have been shown to be effectively passivated by additives,^4^ light soaking^5^ or even by storing under dark and dry conditions.^6–8^ The latter pathway is of particular interest as there are no additional experimental steps or chemicals required. We have shown that the increase in device performance was accompanied by a spontaneous increase in average perovskite grain size, indicating that ions in the perovskite film are very mobile, allowing the perovskite to self-heal as the ions move through the lattice.^6^ It was shown that this ion migration can relax strain present in the perovskite film, eliminating trap states.^7,8^ Moghadamzadeh *et al.* showed that aging-induced performance enhancement plays an important role in a wide range of lead halide perovskite materials and ruled out any influence of the charge transporting materials.^7^

Aging is facilitated by ion migration, which can occur spontaneously, but is greatly enhanced by voltage biasing^9^ and light soaking.^10^ In this study we investigate the influence of the halide ratio on ion migration and track the morphological and optoelectronic properties of aging perovskite films using SEM, XRD, photoluminescence spectroscopy (PL), electrochemical impedance spectroscopy (EIS) and device performance. We chose to use the halide ratio as a lever to modulate ion migration in triple cation perovskite, because previous works have shown that halides (vacancies) are the most mobile species in lead halide perovskites (by 3 orders of magnitude).^11,12^ It was further found that iodide migrates ∼3 times faster than bromide.^13^ This can be explained by the higher bond strength of Pb–Br when compared to Pb–I.^14^ We show that ion migration is essential for self-passivation, but can be impeded by low ion mobility (high bromide content) or a low number of mobile species (pure iodide). One of the most commonly used perovskite composition is the so-called ‘triple cation’ lead-halide perovskite using cesium, methylammonium (MA) and formamidinium (FA) as cations (full composition: Cs_0.05_(MA_0.17_FA_0.83_)_0.95_Pb(I_0.83_Br_0.17_)_3_).^15^ This composition owes its popularity to high efficiency, reproducibility and stability. We find that the 83 : 17 iodide/bromide ratio used in the benchmark triple cation perovskite strikes the perfect balance between the availability and mobility of halide ions to allow efficient self-passivation. This further explains why this particular composition can achieve reliable and reproducible efficiencies. We also find that strained films with impeded ion migration have a tendency to crack, resulting in reduced device performance. As light has been shown to soften the perovskite lattice and enhance ion mobility by weakening ionic bonds,^16^ we explored the use of a short light soaking treatment after fabrication as a tool to enhance ion migration and allow self-passivation and strain relaxation, leading to improved performance for all compositions.

## Results

**Fig. 1:**
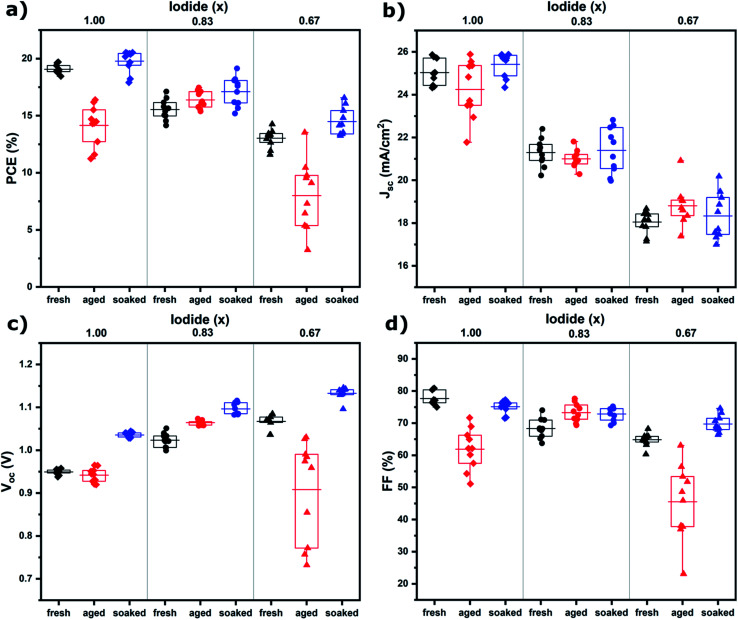
Device parameters of PSCs using Cs_0.05_(MA_0.17_FA_0.83_)_0.95_Pb(I_*x*_Br_1−*x*_)_3_ as the absorber layer, (a) PCE, (b) *J*_sc_, (c) *V*_oc_ and (d) FF, extracted from *J*–*V* scans measured at a 50 mV s^−1^ scan rate under AM1.5 illumination. Each subset contains 10 devices.

The influence of the iodide/bromide ratio on aging was studied by fabricating PSCs using Cs_0.05_(MA_0.17_FA_0.83_)_0.95_Pb(I_*x*_Br_1−*x*_)_3_ as the absorber layer, where *x* is 1.00, 0.83 or 0.67. All devices were aged for 7 days under the same conditions (room temperature, dry air, dark), but one subset was light soaked for 10 minutes shortly after fabrication (100 mW cm^−2^ AM1.5 illumination under ambient conditions). Device parameters (PCE, short circuit current density (*J*_sc_), open circuit potential (*V*_oc_) and fill factor (FF)) of devices after fabrication (fresh), aged for 7 days (aged) and aged for 7 days after initial light soaking (soaked) are shown in Fig. 1, a table containing these values can be found in S1.† It is evident that composition and light soaking have a big influence on the aging behavior of PSCs. For aged devices, an increase in PCE is shown for *x* = 0.83, whereas for *x* = 1.00 and *x* = 0.67 PCE decreases markedly, mainly as a result of a drop in FF. When the devices are aged after light soaking, PCE increases regardless of composition, mainly caused by an increase in *V*_oc_. The performance improvement instigated by light soaking only becomes apparent after aging (S2†). The mechanism is thus different from light-induced oxygen passivation, which has been shown to manifest within minutes.^5^ Hysteresis can hint at the presence of interfacial defects.^17^ The hysteresis index suggests interfacial defects are present in fresh devices, but have largely disappeared after aging and light soaking (S1†).

**Fig. 2:**
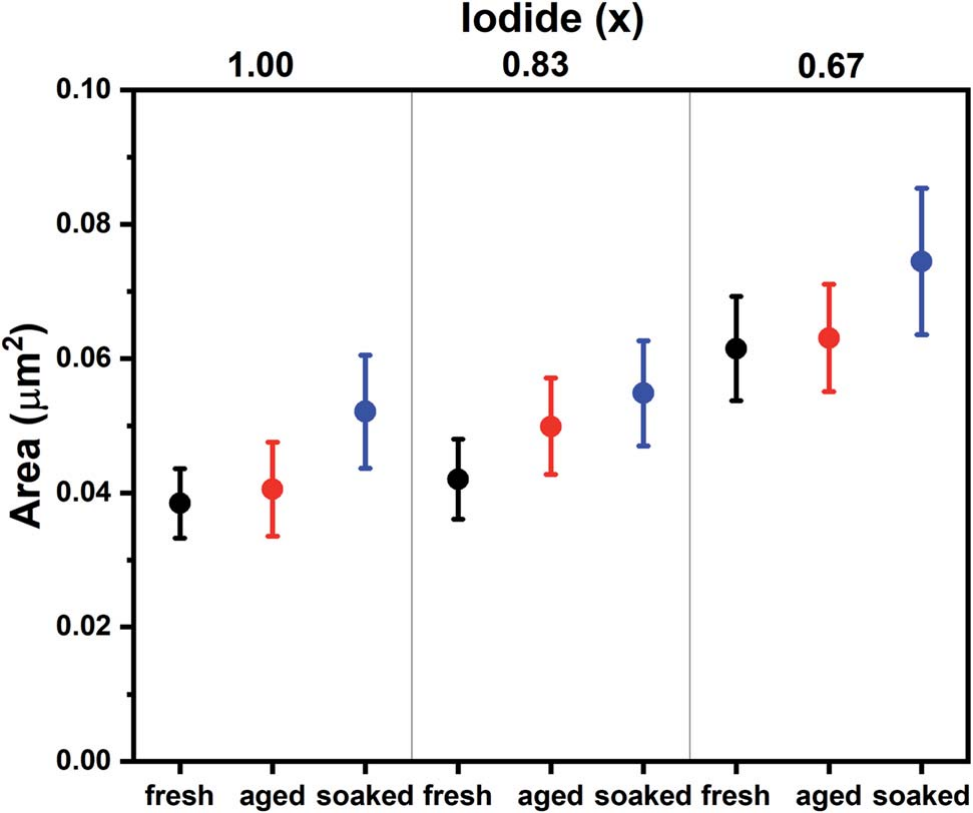
Average grain sizes for Cs_0.05_(MA_0.17_FA_0.83_)_0.95_Pb(I_*x*_Br_1−*x*_)_3_ films as extracted from SEM images (S3†). Whiskers represent the standard error.

To understand the differences between compositions and aged and soaked devices, we employed a range of morphological and optoelectronic characterization techniques. Previous work has shown that morphology is affected by aging and that ions can migrate between grains, resulting in the growth of certain grains at the expense of others.^6^ SEM was employed to monitor changes in morphological grain size upon aging. Grains in SEM are not necessarily crystallographic grains and instead observed grain boundaries can be regions of changing morphology and composition.^18^ SEM images of fresh, aged and soaked devices for *x* = 1.00, 0.87 and 0.67 are shown in S3.† The average grain size extracted from these images is shown in Fig. 2. It is found that under dark conditions the average grain size has increased only for *x* = 0.83. The soaked samples on the other hand all show an increase in average grain size. Interestingly, the change in grain size exhibits the same trend as device performance; under illumination performance and grain size increases for all halide ratios, while in the dark only *x* = 0.83 shows an increase. This suggests that ion migration plays a key role in self-passivation. It is interesting to note that grain growth has been observed by SEM,^6,19^ but not with AFM.^7^ Unlike AFM, SEM detects not only morphology, but chemical composition as well.^20^ This may indicate that a compositional rather than a morphological transformation takes place.

**Fig. 3:**
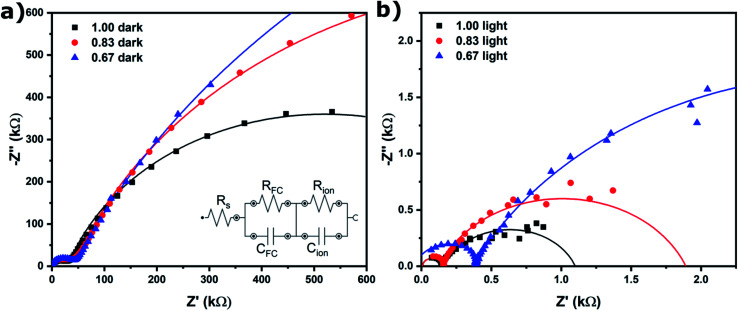
EIS for PSCs using Cs_0.05_(MA_0.17_FA_0.83_)_0.95_Pb(I_*x*_Br_1−*x*_)_3_ as the absorber layer, (a) without illumination (dark) and (b) with illumination (AM1.5, 100 mW cm^−2^). The data was fitted using the equivalent circuit in the inset of (b), where *R*_s_ is the series resistance, *R*_FC_ and *C*_FC_ the free charge carrier recombination resistance and capacitance and *R*_ion_ and *C*_ion_ the ion migration resistance and capacitance.

EIS was performed to quantify ion migration as a function of halide ratio, and both without (Fig. 3a) and with illumination (Fig. 3b) to elucidate the effect of light on ion migration. The low frequency (slow) response in EIS can be attributed to ion migration in the perovskite film (the mid-frequency semi-circle is attributed to migration and recombination of free charge carriers).^21^ The equivalent circuit shown in the inset of Fig. 3a was used to fit the data. The values for the ion migration resistance (*R*_ion_) are shown in Table 1. *R*_ion_ is three orders of magnitude lower under illumination, indicating ions can move much more freely, which is in agreement with the increased grain size found in SEM after light soaking (Fig. 2). This is further proof that ion migration is necessary to facilitate self-passivation and improve device performance (Fig. 1). Light soaking increases ion mobility to such an extent that ion migration is facilitated in all compositions. In addition, *R*_ion_ is found to increase with bromide content, as has been reported in literature^13^ and can be explained by the stronger binding of bromide to its neighboring ions. This is in line with grain size and device performance trends (in the dark) for *x* = 0.83 and *x* = 0.67, showing a significant increase for *x* = 0.83, but in the case of *x* = 0.67 ion mobility is much decreased and no significant ion migration takes place. The only anomalous result is for *x* = 1.00; given the high ion mobility, one would expect to see an increase in grain size and device performance. We will explain this behavior further on by not only taking into account the mobility but also the number of mobile species.

**Table 1:** Values for *R*_ion_ for PSCs using Cs_0.05_(MA_0.17_FA_0.83_)_0.95_Pb(I_*x*_Br_1−*x*_)_3_ as the absorber layer as extracted from the data in Fig. 3. Note that dark *R*_ion_ is in MΩ and light *R*_ion_ in kΩ

Iodide (*x*)	Dark *R*_ion_ (MΩ)	Light *R*_ion_ (kΩ)
1.00	1.01	0.94
0.83	1.93	1.77
0.67	3.29	5.33

**Fig. 4:**
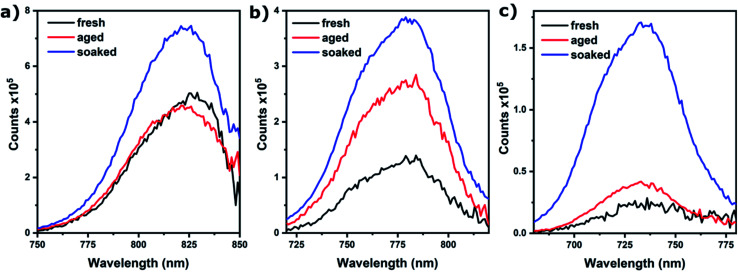
PL intensity for Cs_0.05_(MA_0.17_FA_0.83_)_0.95_Pb(I_*x*_Br_1−*x*_)_3_ films on glass for (a) *x* = 1.00, (b) *x* = 0.83, (c) *x* = 0.67. Excitation wavelength is 500 nm.

Photoluminescence (PL) spectroscopy was used to assess if ion migration, as evidenced by SEM and EIS, indeed leads to passivation. Fig. 4 shows that this is the case; for *x* = 1.00 and 0.67, PL only increases for the soaked samples, whereas for *x* = 0.83 PL increases for both aged and soaked films (but the increase is largest for soaked films). A stronger PL signal generally means there is less non-radiative combination as would be caused by defects in the absorber material.^22^ The fact that an increase of PL is only observed for samples where we also find ion migration, is strong evidence that ion migration is necessary for defect passivation that leads to increased device performance. Another important observation is that PL intensity decreases with increasing bromide content. This is an important indicator of the mechanism behind ion migration in these materials and provides a possible explanation for the anomalous behavior of the device performance and grain growth for *x* = 1.00 films aged without light soaking. As this composition is relatively defect free, and ion migration is mainly driven by defects (such as vacancies), no grain growth or improved performance is observed, despite the high mobility of ions in this material.

**Fig. 5:**
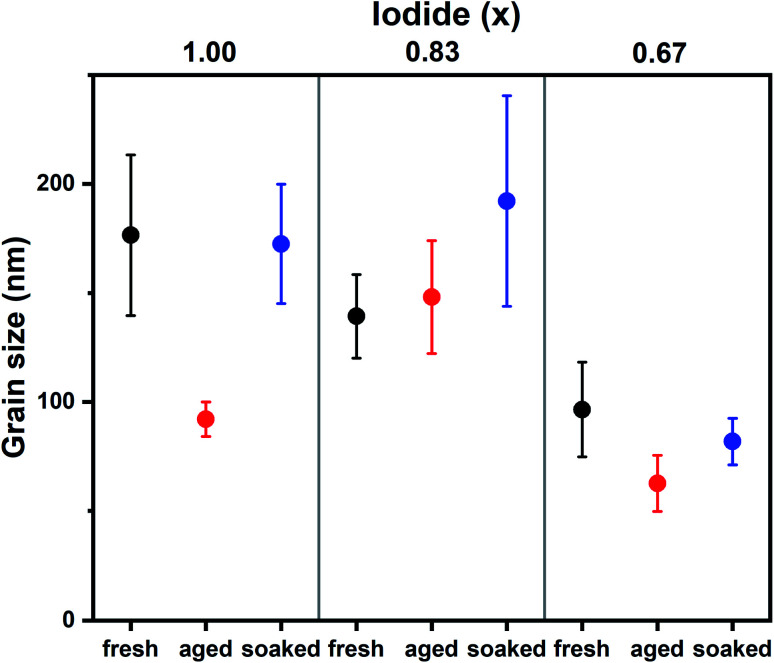
Average grain size for Cs_0.05_(MA_0.17_FA_0.83_)_0.95_Pb(I_*x*_Br_1−*x*_)_3_ films as extracted from XRD data (S4†).

We have now shown that ion migration is the driving force behind aging induced performance enhancement and that if ion migration is impeded, material properties do not improve. However, this does not explain the drop in FF for devices in which ion migration is restricted (*x* = 1.00 aged and *x* = 0.67 aged). For this we have to take strain into consideration. During formation, perovskite films are annealed at elevated temperatures. This can lead to a tensile strain in the film upon cooling down (provided the thermal expansion coefficient of the substrate is smaller than that of perovskite, which is the case here, substrates are glass and TiO_2_).^23^ Ion migration can help to relax this tensile strain by moving ions towards strained areas. When ion migration is impeded, strain can cause the formation of cracks in the perovskite film. SEM images do not show any evidence of cracking, but as strain will be manifested at the perovskite/substrate interface and SEM only probes the top surface, a different characterization technique is needed to detect crack formation. We used XRD to track the grain size throughout the perovskite film, expecting the average grain size to decrease when cracks are formed. Fig. 5 shows average grain sizes for fresh, aged and soaked films (see S4† for full diffractograms). The most striking changes are the decreased grain sizes for the aged *x* = 1.00 and *x* = 0.67. This is a strong indication that tensile strain can cause perovskite films to crack at the strained interface when ion migration is impeded, leading to decreased device performance. The trend in the evolution of tensile strain obtained from Williamson–Hall analysis generally shows decreased tensile strain for aged and soaked films (S5†). The fact that strain is released either through ion migration or by cracking can further explain the lower hysteresis index after aging regardless of composition. The films that were subjected to a short light soaking treatment after fabrication do not show a significant reduction in grain size, indicating that light soaking is an effective measure to relax strain. As strain is almost impossible to avoid in annealed films, all perovskite compositions can benefit from a short light soaking treatment shortly after fabrication. We advise the community to adopt a short light soaking treatment after fabrication as a standard procedure in order to improve efficiency and reproducibility.

## Conclusions

We tracked the morphological and optoelectronic properties of triple cation perovskite films and devices under different aging conditions and found that there are significant differences depending on the ratio of the halide ions. The halide ratio used in the benchmark triple cation (*x* = 0.83) leads to improved device performance without external stimuli. However, for *x* = 1.00 and *x* = 0.67 a short light soaking treatment before aging is necessary to achieve self-passivation. SEM and EIS measurements show that this behavior is closely related to ion migration in the perovskite film. As ions migrate through the film they heal defects and relax strain. When ion migration is impeded, such as by strong ionic binding for *x* = 0.67, or by a low number of mobile species for *x* = 1.00, cracks can form in the perovskite film. This has a detrimental effect on device performance, but a short light soaking treatment sufficiently increases ion migration to mitigate this problem. These results can help to explain the success of the benchmark triple cation perovskite. But as materials with less mobile species (*i.e.* compositions with few intrinsic defects for high efficiency devices) or low ion mobility (with higher bromide content for bandgap tuning) are become more popular, it is important for the community to note that these materials greatly benefit from a short light soaking treatment shortly after fabrication, both in terms of efficiency and reproducibility. We propose a short light soaking treatment to be adopted as a routine procedure.

## Methods

### Materials

Methylammonium bromide, formamidinium iodide and titania paste (30NR-D) were purchased from Greatcell Solar, PbI_2_ and PbBr_2_ from TCI and spiro-OMeTAD from Borun Technology. All other chemicals were purchased from Sigma Aldrich.

### Solar cell fabrication

Fluorine doped tin oxide coated glass was cleaned by sonication in 2% Hellmanex III solution for 15 minutes, rinsed with deionized water and sonication in isopropanol for 15 minutes. Substrates were dried and transferred to a hotplate and heated to 450 °C. Compact TiO_2_ was deposited by spray pyrolysis of a solution containing 9 ml ethanol, 0.6 ml titanium(iv) diisopropoxide bis(acetylacetonate) and 0.4 ml acetylacetone. Substrates were cooled to room temperature before the mesoporous TiO_2_ (150 mg ml^−1^ paste in ethanol) was deposited by spincoating (4000 rpm, 10 s, 2000 rpm ramp). After spincoating the substrates were transferred to a hotplate preheated at 125 °C and the following protocol was used for annealing: 10 min at 125 °C, 15 min ramp and 5 min dwell at 325 °C, 5 min ramp and 5 min dwell at 375 °C and 5 min ramp and 30 min dwell at 450 °C. Substrates were then allowed to cool to 150 °C, after which they were transferred to a N_2_ filled glovebox for perovskite deposition. Perovskite films were deposited from a precursor solution containing, *x* = 1.00: FAI (1 M), PbI_2_ (1.32 M), MAI (0.2 M) and CsI (0.075 M), *x* = 0.83: FAI (1 M), PbI_2_ (1.1 M), MABr (0.2 M), PbBr_2_ (0.22 M) and CsI (0.075 M), *x* = 0.67: FAI (1 M), PbI_2_ (0.77 M), MABr (0.2 M), PbBr_2_ (0.55 M) and CsI (0.075 M) in anhydrous DMF : DMSO 4 : 1 (v/v). The perovskite solution was spin-coated in a two-step program at 1000 and 6000 rpm for 10 and 20 s respectively. During the second step, 100 μl of chlorobenzene was poured onto the spinning substrate 5 seconds prior the end of the program. The substrates were then transferred to a hotplate preheated to 100 °C and annealed for 60 min.^15^ After cooling down to room temperature, spiro-OMeTAD (0.07 M) in chlorobenzene, doped with *t*-butylpyridine (3.3 mol mol^−1^), bis(trifluoromethane)sulfonamide lithium (0.5 mol mol^−1^) and tris(2-(1*H*-pyrazol-1-yl)-4-*tert*-butylpyridine)-cobalt(iii)tris(bis(trifluoromethylsulfonyl)imide) (0.05 mol mol^−1^) was deposited by spincoating (4000 rpm, 20 s). Devices were finished by thermal evaporation of 60 nm gold.

### Optoelectronic characterization

A solar simulator from ABET Technologies (Model 11016 Sun 2000) with a xenon arc lamp was used to illuminate the solar cells for *J*–*V*-measurements (100 mW cm^−2^, AM1.5) which were recorded using a Keithley 2635 sourcemeter. *J*–*V* measurements were recorded at a scan speed of 50 mV s^−1^ from open circuit to short circuit conditions.

PL measurements were performed using a 450 W continuous xenon arc lamp and an Edinburgh Instruments FLS980 fluorimeter. During the measurements, the excitation wavelength was fixed at 500 nm, with a wavelength step of 1 nm and dwell time of 1 s.

### Scanning electron microscopy

Scanning electron microscopy was performed using a Zeiss LEO 1550 FE-SEM with a field emission source operating at 2 kV acceleration voltage in the In-Lens mode.

### Electrochemical impedance spectroscopy

EIS was performed using a Metrohm PGSTAT302N Autolab. Spectra were recorded at a frequency range of 100 kHz to 0.1 Hz and fitted using Nova 1.12 software.

### X-ray diffraction

X-ray diffractograms of perovskite films were obtained in Bragg–Brentano geometry using a Bruker D8 Advance X-ray diffractometer with CuKα radiation (*λ* = 1.5418 Å). All the measurements were performed with 2*θ* angles ranging from 10° to 40°, with a step size of 0.00214°.

## Conflicts of interest

There are no conflicts of interest to declare

## Supplementary Material

RA-011-D1RA01166A-s001
