# Supplementary material for: Ion migration drives self-passivation in perovskite solar cells and is enhanced by light soaking
Source: RSC Adv. 2021 Mar 25;11(20):12095–101. doi: 10.1039/d1ra01166a (PMC8696990; doi:10.1039/d1ra01166a)
Supplement: RA-011-D1RA01166A-s001 [file RA-011-D1RA01166A-s001.pdf]

Electronic Supplementary Material (ESI) for RSC Advances.
This journal is © The Royal Society of Chemistry 2021

Supporting Information

Ion Migration Drives Self-Passivation in Perovskite Solar Cells and is Enhanced by Light Soaking

Bart Roose[1]

[1] *Department of Physics, Cavendish Laboratory, University of Cambridge, 19 JJ Thomson Avenue, Cambridge, CB3 0HE, UK*

S1. Device parameters of perovskite solar cells with varying halide ratio and aging conditions. Hysteresis index is defined as $(PCE_{backwards}-PCE_{forwards})/(PCE_{backwards}+PCE_{forwards})$. Averages of 10 devices.

Iodide ratio	Condition	V_{oc} (mV)	J_{sc} (mA/cm^2)	FF (%)	PCE (%)	Hysteresis index
**1**	**fresh**	949 ± 6	25.0 ± 0.6	78 ± 2	19.1 ± 0.4	0.12 ± 0.03
	**aged**	942 ± 17	24.2 ± 1.3	62 ± 6	14.1 ± 1.8	0.02 ± 0.02
	**soaked**	1035 ± 6	25.4 ± 0.6	75 ± 2	19.8 ± 1.1	0.02 ± 0.02
**0.83**	**fresh**	1023 ± 16	21.3 ± 0.6	68 ± 3	15.5 ± 0.9	0.12 ± 0.03
	**aged**	1064 ± 5	21.0 ± 0.4	73 ± 3	16.4 ± 0.7	0.01 ± 0.02
	**soaked**	1096 ± 13	21.4 ± 1.1	73 ± 2	17.1 ± 1.3	0.01 ± 0.02
**0.67**	**fresh**	1067 ± 18	18.0 ± 0.5	65 ± 2	13.0 ± 0.8	0.08 ± 0.01
	**aged**	842 ± 201	18.7 ± 0.6	44 ± 10	7.0 ± 2.7	0.01 ± 0.01
	**soaked**	1133 ± 14	18.3 ± 1.1	70 ± 3	14.5 ± 1.2	0.02 ± 0.03

S2. Device parameters of perovskite solar cells directly before and after 10 minutes of light soaking.

Condition	V_{oc} (mV)	J_{sc} (mA/cm^2)	FF (%)	PCE (%)
**before soak**	1007 ± 18	21.9 ± 0.1	76 ± 1	16.8 ± 0.5
**after soak**	1012 ± 24	21.7 ± 0.1	76 ± 2	16.7 ± 0.7

S3. SEM images of $Cs_{0.05}(MA_{0.17}FA_{0.83})_{0.95}Pb(I_xBr_{1-x})_3$ perovskite films (x=1.00, 0.83 and 0.67), just after fabrication (fresh), after aging for 7 days in dark and dry conditions (aged) and after aging following a ten-minute light soaking treatment (soaked). Scale bar is 400 nm.

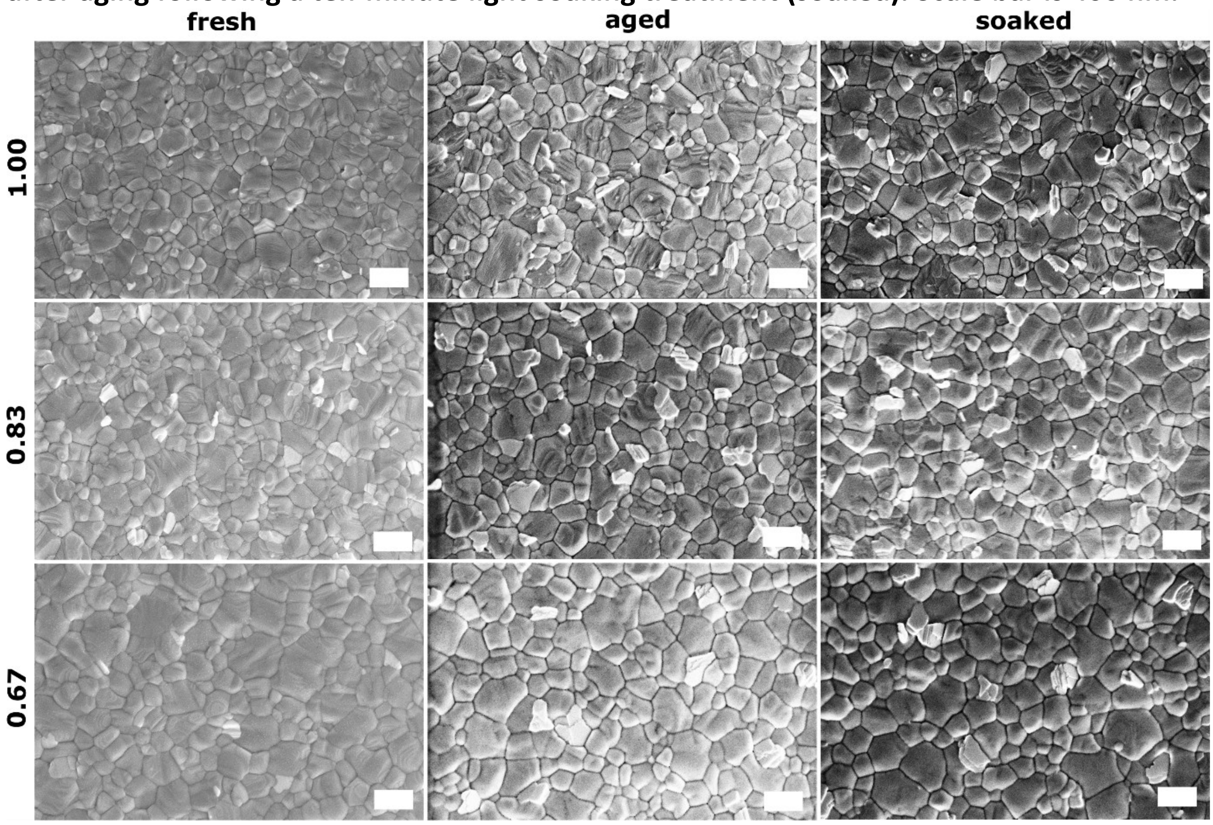

S4. X-ray diffractograms of $Cs_{0.05}(MA_{0.17}FA_{0.83})_{0.95}Pb(I_xBr_{1-x})_3$ perovskite films (x=1.00, 0.83 and 0.67), just after fabrication (fresh), after aging for 7 days in dark and dry conditions (aged) and after aging following a ten-minute light soaking treatment (soaked). Peaks labeled with a star stem from the FTO substrate.

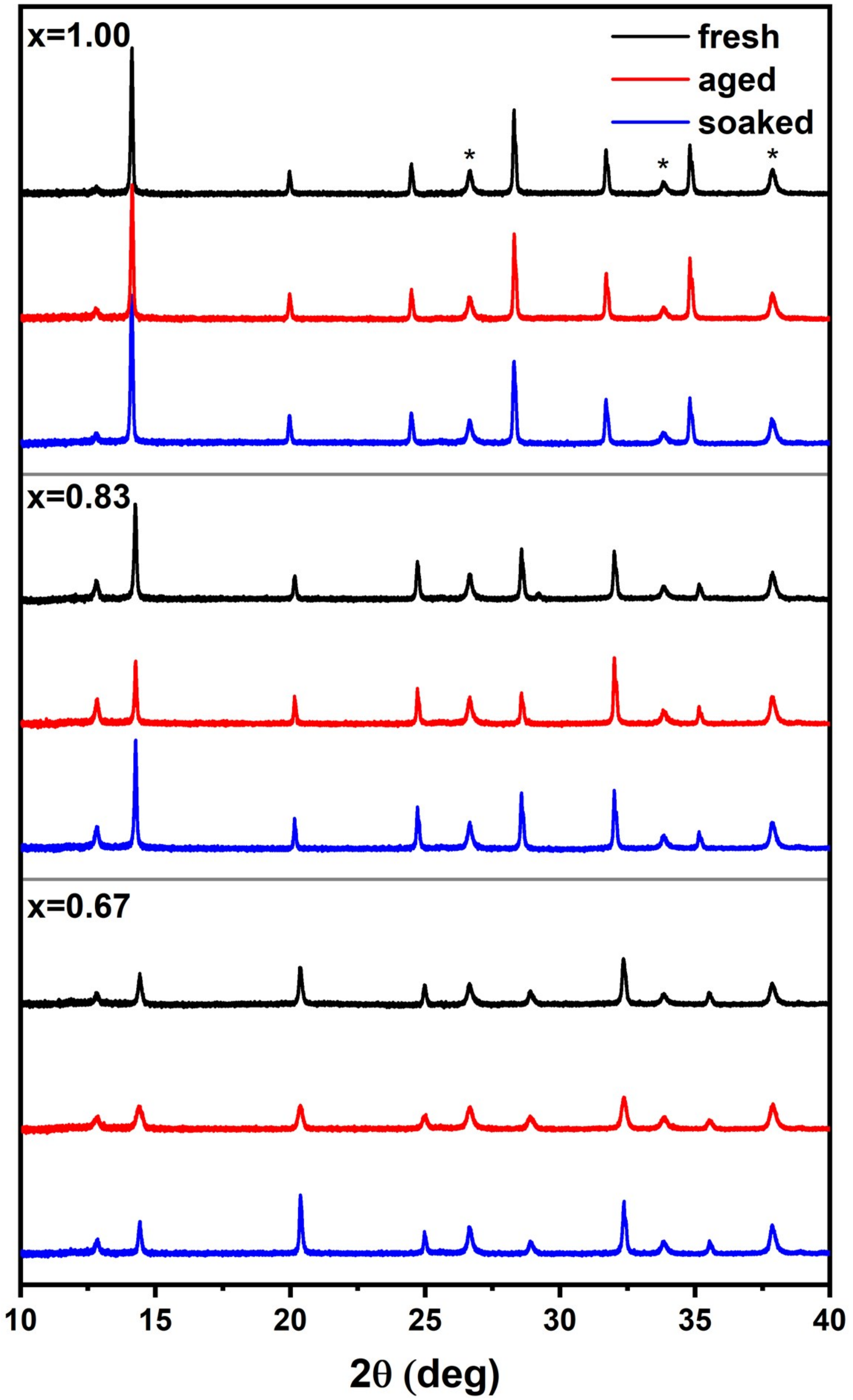

S5. Williamson-Hall analysis of $Cs_{0.05}(MA_{0.17}FA_{0.83})_{0.95}Pb(I_xBr_{1-x})_3$ perovskite films (x=1.00, 0.83 and 0.67), just after fabrication (fresh), after aging for 7 days in dark and dry conditions (aged) and after aging following a ten-minute light soaking treatment (soaked) and corresponding strain values (Cε).

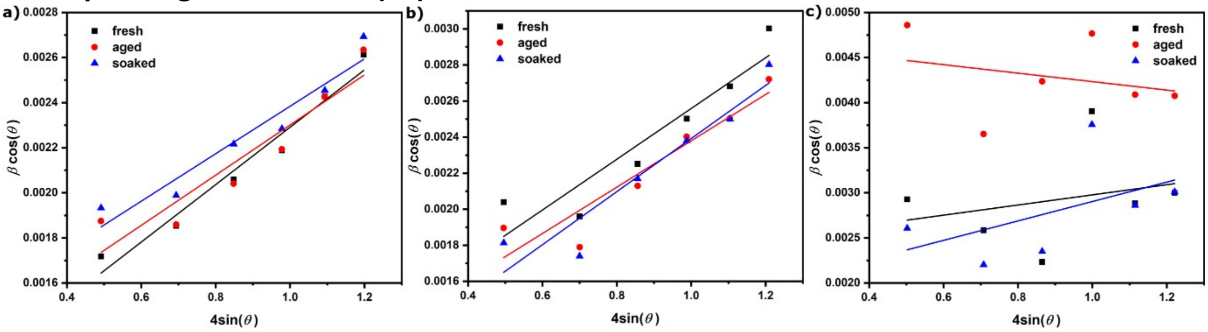

Iodide ratio	Condition	Cε (10^-3)
**1**	fresh	1.3 ± 0.1
	aged	1.1 ± 0.2
	soaked	1.1 ± 0.2
**0.83**	fresh	1.4 ± 0.3
	aged	1.3 ± 0.2
	soaked	1.5 ± 0.3
**0.67**	fresh	0.6 ± 1.0
	aged	-0.5 ± 0.8
	soaked	1.1 ± 0.9
